# Reemergence of *Bordetella parapertussis*, United States, 2019–2023

**DOI:** 10.3201/eid3005.231278

**Published:** 2024-05

**Authors:** Brooklyn A. Noble, Sarah S. Jiudice, Jay D. Jones, Tristan T. Timbrook

**Affiliations:** bioMérieux, Salt Lake City, Utah, USA (B.A. Noble, S.S. Jiudice, J.D. Jones, T.T. Timbrook);; University of Utah, Salt Lake City (T.T. Timbrook)

**Keywords:** Bordetella parapertussis, Bordetella pertussis, whooping cough, surveillance network, public health, bacteria, COVID-19, 2019 novel coronavirus disease, coronavirus disease, severe acute respiratory syndrome coronavirus 2, SARS-CoV-2, viruses, respiratory infections, zoonoses, United States

## Abstract

To determine changes in *Bordetella pertussis* and *B. parapertussis* detection rates, we analyzed 1.43 million respiratory multiplex PCR test results from US facilities from 2019 through mid-2023. From mid-2022 through mid-2023, *Bordetella* spp. detection increased 8.5-fold; 95% of detections were *B. parapertussis.* While *B. parapertussis* rates increased, *B. pertussis* rates decreased.

Whooping cough is a highly contagious, acute, respiratory illness caused by *Bordetella* spp. bacteria, primarily *B. pertussis* and *B. parapertussis*, and may be associated with complications such as pneumonia (*1*,*2*). Unlike *B. pertussis*, *B. parapertussis* is not notifiable in the United States because it is thought to be less prevalent and to cause milder symptoms than *B. pertussis* (*1*,*2*). Although isolation of *B. parapertussis* was uncommon in the United States before 2005, it has since been suggested that *B. parapertussis* infections are more common than previously recognized and may contribute to cases thought to result from vaccine failure (*1*,*3*). Our objective with this study was to detect recent changes in *B. pertussis* and *B. parapertussis* detection rates by using a cloud-based near real-time surveillance network.

We analyzed >1.43 million multiplex PCR results from 125 US facilities for January 1, 2019–July 31, 2023, for detection of *B. pertussis* or *B. parapertussis* (Table). Information on clinical manifestations, patient demographics, and confirmatory testing were not known. Facilities were primarily reference laboratories or hospitals, 12 of which were pediatric or contained a pediatric site, and all facilities used the BIOFIRE FILMARRAY Respiratory 2 (RP2) Panel, the BIOFIRE Respiratory 2.1 (RP2.1) Panel (bioMérieux, https://www.biomerieux.com), or both (*4*,*5*). The RP2 and RP2.1 tests detect nucleic acid of 21 (RP2) or 22 (RP2.1) pathogens commonly associated with respiratory infections and are identical, except the RP2.1 test can also detect SARS-CoV-2. Both tests detect *B. pertussis* (limit of detection of 1.0 × 10^3^ CFU/mL) and *B. parapertussis* (limit of detection 4.1 × 10^1^ CFU/mL) (*5*). Deidentified patient test results were captured by the BIOFIRE Syndromic Trends database, a cloud-based pathogen surveillance network (*6*). We excluded suspected verification, quality control, and proficiency tests (*6*).

**Table:** *Bordetella parapertussis* testing, United States, 2019–2023*

Year, region	States	No. (%) tests performed		
		Facilities	RP2 tests	RP2.1 tests†
2019				
Midwest	IL, IN, KS, MI, MO, ND, NE, OH, SD, WI	19 (52.8)	49,226 (48.9)	0
Northeast	NY, PA	3 (8.3)	6,252 (6.2)	0
South	FL, SC, TX	6 (16.7)	13,109 (13.0)	0
West	AK, AZ, CA, ID, UT	8 (22.2)	31,989 (31.8)	0
2020				
Midwest	IL, IN, KS, MI, MO, ND, NE, OH, SD, WI	36 (45.0)	44,545 (27.3)	44,832 (27.5)
Northeast	NY, PA	5 (6.2)	1,988 (1.2)	1,511 (0.9)
South	AL, FL, GA, LA, SC, TN, TX, VA	18 (22.5)	9,208 (5.6)	17,352 (10.6)
West	AK, AZ, CA, CO, ID, OR, UT, WY	20 (25.0)	25,663 (15.7)	13,845 (8.5)
2021				
Midwest	IA, IL, IN, KS, MI, MO, ND, NE, OH, SD, WI	40 (39.2)	3125 (0.8)	137,322 (36.8)
Northeast	MA, NY, PA	6 (5.9)	0	37,207 (10.0)
South	AL, AR, FL, GA, KY, LA, MD, MS, SC, TN, TX, VA	31 (30.4)	405 (0.1)	124,544 (33.4)
West	AK, AZ, CA, CO, ID, MT, NM, OR, UT, WY	25 (24.5)	1,709 (0.5)	64,733 (17.4)
2022				
Midwest	IA, IL, IN, KS, MI, MO, ND, NE, OH, SD, WI	41 (36.9)	502 (0.1)	169,739 (33.9)
Northeast	MA, NJ, NY, PA, VT	8 (7.2)	0	65,602 (13.1)
South	AL, AR, FL, GA, KY, LA, MD, MS, NC, SC, TN, TX, VA	35 (31.5)	0	174,014 (34.8)
West	AK, AZ, CA, CO, ID, MT, NM, OR, UT, WA, WY	26 (23.4)	221	87,262 (17.5)
2023‡				
Midwest	IA, IL, IN, KS, MI, MO, ND, NE, OH, SD, WI	39 (34.8)	0	98,274 (31.7)
Northeast	MA, NJ, NY, PA, VT	8 (7.1)	0	51,160 (16.5)
South	AL, AR, FL, GA, KY, LA, MD, MS, NC, SC, TN, TX, VA	37 (33.0)	0	114,433 (36.9)
West	AK, AZ, CA, CO, ID, MT, NM, OR, UT, WA, WY	27 (24.1)	0	44,612 (14.4)

*Rates consider the total number for each year across all regions and test versions. †The BIOFIRE RP2.1 test (bioMérieux, https://www.biomerieux-usa.com, authorized for emergency use in May 2020) is identical to the BIOFIRE RP2 test except for the addition of SARS-CoV-2. Use of the 2 test versions overlapped primarily during June–July 2020. During that time, comparison of detection rates of the 2 test versions did not show strong evidence of a statistically significant difference for *B. pertussis* (p = 0.16) or *B. parapertussis* (p = 0.72). ‡The 2023 data collection period was January–July.

We determined the total number of tests in the database and the number of those tests that detected *B. pertussis* or *B. parapertussis*, aggregated monthly (Figure, panel A), and detection rates (3-week centered rolling average) for *B. pertussis* and *B. parapertussis* (stacked) (Figure, panel B). During January 1, 2019–March 11, 2020 (before the COVID-19 pandemic), we observed that in the United States, the average (95% binomial CI) *B. pertussis* detection rates (0.14% [95% CI 0.12%–0.16%]) were slightly lower than the *B. parapertussis* detection rates (0.21% [95% CI 0.18%–0.23%]). From mid-2020 through late 2022, the detection rates of *B. pertussis* and *B. parapertussis* declined significantly; the combined rate remained <0.20%. In 2023 (January–July), we observed a marked increase in *B. parapertussis* detections; average detection rate was 0.65% (95% CI 0.62%–0.68%) and peaked mid-June at 1.3% (95% CI 1.1%–1.6%). We did not observe a similar increase in *B. pertussis* detections, for which the average detection rate in 2023 was 0.03% (95% CI 0.02%–0.04%).

**Figure:**
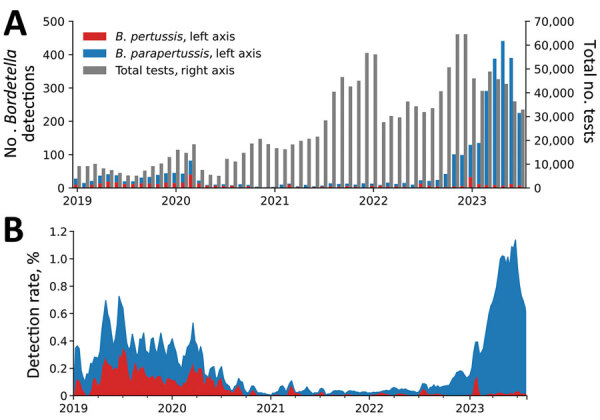
*Bordetalla pertussis* and *B. parapertussis* detection count and detection rates, January 2019–July 2023. A) Total number of tests and number of tests positive for *B. pertussis* or *B. parapertussis* per month. Scales for the y-axes differ substantially to underscore patterns but do not permit direct comparisons. B) Detection rate (3-week centered rolling average) for *B. pertussis* and *B. parapertussis*.

Comparing recent (January 2023–July 2023) rates to rates from a commensurate prepandemic time frame (January 2019–July 2019), we observed an increase of 0.44% (95% CI 0.39%–0.49%) for *B. parapertussis* and a decrease of 0.12% (95% CI 0.09%–0.16%) for *B. pertussis*. Those findings lend evidence to a significant (p<0.001) national *B. parapertussis* increase and *B. pertussis* decrease; similar trends were observed in each US Census region. Of the 23 facilities with data for both time frames, the *B. parapertussis* detection rate increased for 20 facilities.

Co-detection of *B. pertussis* and *B. parapertussis* in the same test was rare, observed in only 9 tests (0.03% of tests positive for either *B. pertussis* or *B. parapertussis*) (Appendix Table). However, a virus was co-detected with 47.1% (95% CI 42.5%–51.7%) of *B. pertussis* and 66.2% (95% CI 64.4%–68.0%) of *B. parapertussis* detections.

In summary, we found that 95% of *Bordetella* spp. detected in the last year of the study (July 2022– July 2023) were *B. parapertussis*. The observed high incidence of virus co-detections along with previous data that found that clinical infection developed in <5% of those with *B. parapertussis* (compared with 75% of those with *B. pertussis*) may suggest that many of the observed *B. parapertussis* detections were subclinical (*7*). Although the reason behind the observed increase in *B. parapertussis* detections is unknown, Bhattacharyya et al. suggested that the erratic dynamics of whooping cough could be explained by interactions of *B. pertussis* and *B. parapertussis*, which oscillate out of phase through age-dependent convalescence (*8*). It is possible that secondary effects of the COVID-19 pandemic, such as decreased population immunity, affected this interaction, because incidence of many other respiratory illnesses also decreased during the pandemic, followed by atypical prevalence (*9*).

Testing and near real-time surveillance of *B. parapertussis* are needed to enhance prompt response to clinical outbreaks and contamination events, both of which have been reported (*1*,*10*). Determining the clinical implications of the observed *B. parapertussis* surge may help inform patient management and public health action.

Appendix Additional results for study of reemergence of *Bordetella parapertussis*, United States, 2019–2023.
