# Supplementary material for: Reemergence of Bordetella parapertussis, United States, 2019–2023
Source: Emerg Infect Dis. 2024 May;30(5):1058–60. doi: 10.3201/eid3005.231278 (PMC11060467; doi:10.3201/eid3005.231278)
Supplement: Appendix — Additional results for study of reemergence of Bordetella parapertussis, United States, 2019–2023. [file 23-1278-Techapp-s1.pdf]

Article DOI: https://doi.org/10.3201/eid3005.231278

EID cannot ensure accessibility for supplementary materials supplied by authors. Readers who have difficulty accessing supplementary content should contact the authors for assistance.

Reemergence of *Bordetella parapertussis*, United States, 2019–2023

Appendix

Appendix Table. Detection, *Bordetella pertussis* co-detection, and *Bordetella parapertussis* co-detection counts and rates for each pathogen included on the multiplex PCR tests used in study of reemergence of *B. parapertussis*, United States, 2019–2023*

	No. (%)		
Pathogen	Detection	*B. pertussis* codetection	*B. parapertussis* codetection
Viruses			
Adenovirus	61,058 (4.2)	53 (11.1)	373 (13.7)
Coronavirus (not SARS-CoV-2)	47,519 (3.3)	21 (4.4)	151 (5.5)
Coronavirus SARS-CoV-2†	126,879 (10.1)	9 (1.9)	46 (1.7)
Human metapneumovirus	44,057 (3.0)	11 (2.3)	116 (4.3)
Human rhinovirus/enterovirus	267,749 (18.5)	127 (26.6)	1,039 (38.1)
Influenza A	45,690 (3.2)	10 (2.1)	95 (3.5)
Influenza B	5,710 (0.4)	11 (2.3)	29 (1.1)
Parainfluenza virus	69,436 (4.8)	19 (4.0)	264 (9.7)
Respiratory syncytial virus	76,609 (5.3)	26 (5.4)	96 (3.5)
Bacteria			
B. parapertussis	2,724 (0.2)	9 (1.9)	NA
B. pertussis	478 (0.0)	NA	9 (0.3)
Chlamydia pneumoniae	848 (0.1)	2 (0.4)	14 (0.5)
Mycoplasma pneumoniae	2,106 (0.1)	2 (0.4)	9 (0.3)

*Detection rates consider all tests. *B. pertussis* co-detection rates consider only tests in which *B. pertussis* was detected, and *B. parapertussis* co-detection rates consider only tests in which *B. parapertussis* was detected. NA, not applicable.
†Only the BIOFIRE RP2.1 test (bioMérieux, https://www.biomerieux-usa.com) was able to detect SARS-CoV-2.
